# Social Equity in the Efficacy of Computer-Based and In-Person Brief Alcohol Interventions Among General Hospital Patients With At-Risk Alcohol Use: A Randomized Controlled Trial

**DOI:** 10.2196/31712

**Published:** 2022-01-28

**Authors:** Jennis Freyer-Adam, Sophie Baumann, Gallus Bischof, Andreas Staudt, Christian Goeze, Beate Gaertner, Ulrich John

**Affiliations:** 1 Institute for Medical Psychology University Medicine Greifswald Greifswald Germany; 2 German Centre for Cardiovascular Research (DZHK) Greifswald Germany; 3 Department of Methods in Community Medicine Institute of Community Medicine University Medicine Greifswald Greifswald Germany; 4 Department of Psychiatry and Psychotherapy Medical University of Lübeck Luebeck Germany; 5 Institute and Policlinic of Occupational and Social Medicine Faculty of Medicine Technische Universität Dresden Dresden Germany; 6 Department of Epidemiology and Health Monitoring Robert Koch Institute Berlin Berlin Germany; 7 Department of Prevention Research and Social Medicine Institute of Community Medicine University Medicine Greifswald Greifswald Germany

**Keywords:** brief alcohol intervention, electronic, eHealth, digital, motivational interviewing, socioeconomic status, equity, social inequality, transtheoretical model, moderator, mental health, public health, alcohol interventions, digital intervention, digital health intervention, alcohol use

## Abstract

**Background:**

Social equity in the efficacy of behavior change intervention is much needed. While the efficacy of brief alcohol interventions (BAIs), including digital interventions, is well established, particularly in health care, the social equity of interventions has been sparsely investigated.

**Objective:**

We aim to investigate whether the efficacy of computer-based versus in-person delivered BAIs is moderated by the participants’ socioeconomic status (ie, to identify whether general hospital patients with low-level education and unemployed patients may benefit more or less from one or the other way of delivery compared to patients with higher levels of education and those that are employed).

**Methods:**

Patients with nondependent at-risk alcohol use were identified through systematic offline screening conducted on 13 general hospital wards. Patients were approached face-to-face and asked to respond to an app for self-assessment provided by a mobile device. In total, 961 (81% of eligible participants) were randomized and received their allocated intervention: computer-generated and individually tailored feedback letters (CO), in-person counseling by research staff trained in motivational interviewing (PE), or assessment only (AO). CO and PE were delivered on the ward and 1 and 3 months later, were based on the transtheoretical model of intentional behavior change and required the assessment of intervention data prior to each intervention. In CO, the generation of computer-based feedback was created automatically. The assessment of data and sending out feedback letters were assisted by the research staff. Of the CO and PE participants, 89% (345/387) and 83% (292/354) received at least two doses of intervention, and 72% (280/387) and 54% (191/354) received all three doses of intervention, respectively. The outcome was change in grams of pure alcohol per day after 6, 12, 18, and 24 months, with the latter being the primary time-point of interest. Follow-up interviewers were blinded. Study group interactions with education and employment status were tested as predictors of change in alcohol use using latent growth modeling.

**Results:**

The efficacy of CO and PE did not differ by level of education (*P*=.98). Employment status did not moderate CO efficacy (*P*s≥.66). Up to month 12 and compared to employed participants, unemployed participants reported significantly greater drinking reductions following PE versus AO (incidence rate ratio 0.44, 95% CI 0.21-0.94; *P*=.03) and following PE versus CO (incidence rate ratio 0.48, 95% CI 0.24–0.96; *P*=.04). After 24 months, these differences were statistically nonsignificant (*P*s≥.31).

**Conclusions:**

Computer-based and in-person BAI worked equally well independent of the patient’s level of education. Although findings indicate that in the short-term, unemployed persons may benefit more from BAI when delivered in-person rather than computer-based, the findings suggest that both BAIs have the potential to work well among participants with low socioeconomic status.

**Trial Registration:**

ClinicalTrials.gov NCT01291693; https://clinicaltrials.gov/ct2/show/NCT01291693

## Introduction

People with low socioeconomic status (SES) have a greater risk of cancer, cardiovascular, and all-cause mortality [1]. Social inequality in health and mortality is increasing [2-4], and alcohol-related mortality plays a crucial role [5]. People with low SES have a 1.7-fold increased risk of dying from alcohol-attributable causes [6]. Alcohol-related causes are responsible for 5% of social inequality in total mortality in European men aged 35 to 79 years, and in some Eastern and Northern European countries, they account for 10% or more [7]. In addition, SES moderates the effect of alcohol use on harm (ie, even when alcohol use is uniform, alcohol-attributable harm is greater in people with low SES [8]).

To close the social inequity gap, behavior change interventions need positive social equity impact (ie, greater reach and greater efficacy in low vs high SES people [5]). To prevent the further widening of the social inequality gap, interventions need neutral impact (ie, equal reach and equal efficacy in low and high SES people). Interventions with greater reach and greater efficacy in high than in low SES people have a negative social equity impact. As reach and efficacy constitute two dimensions of the public health impact of interventions [9], achieving positive or neutral social equity impact at least is a crucial challenge for preventive efforts directly targeting behavior change on the population level.

However, while effective brief alcohol interventions (BAI) have been developed as supported by numerous systematic reviews and meta-analyses [10-15], research findings on the social equity impact of BAI are less encouraging. Firstly, intervention trials, including our own, often report a lower reach of people with low SES or low education, an SES indicator [16,17]. Secondly, little research has been done on the moderating effects of SES indicators, such as level of education and employment status, on intervention efficacy in general. Particularly, little is known about the effect of unemployment status [18]. Thirdly, in some studies, efficacy was found to be reduced in people with lower levels of education than in people with higher levels of education [19,20], indicating that behavior change interventions may have a negative impact on social equity. Reviews revealed a neutral impact once the participants had been recruited [17,21].

Moreover, the development of digital behavior change interventions is advancing. Computer-based interventions have been found to reduce alcohol use in health care [22-24] and beyond [21,25-28]. As they require fewer resources than in-person delivered interventions, their potential impact on public health and social equity may be considered high. Among general hospital patients, our research group showed that computer-based BAI was no less effective than in-person BAI in reducing alcohol use and improving measures of health over two years [29-31]. Thus, computer-based BAI appears to be incorporable into a broader health care program. However, little is known about whether computer-based and in-person delivered interventions work differently for people with low versus high SES.

The aim of this study was to investigate two indicators of SES as moderators of BAI efficacy, namely level of education and employment status. Specifically, we aimed to investigate 3 questions: (1) Does the efficacy of computer-based BAI differ between persons with low versus high levels of education and between unemployed versus employed persons? (2) Does the efficacy of in-person BAI differ between persons with low versus high levels of education and between unemployed versus employed persons? (3) Does the comparative efficacy of computer-based versus in-person BAI differ between persons with low versus high levels of education and between unemployed versus employed persons?

## Methods

### Overview

The data used for these analyses are from the three-arm randomized controlled trial (RCT) entitled “Testing delivery channels of individualized motivationally tailored alcohol interventions among general hospital patients: in-person versus computer-based, PECO” (ClinicalTrials.gov: NCT01291693). The local ethics committee approved the study (BB 07/10, BB 05/13), and the study was conducted as planned.

Sample recruitment took place from February 2011 to July 2012 on four medical departments (internal medicine, surgical medicine, trauma surgery, and ear-nose-throat wards) of the University Medicine Hospital Greifswald [16,31]. All consecutively admitted patients aged 18 to 64 years were first approached face-to-face and asked to respond to an app for self-assessment of health behaviors provided by a mobile device. Patients were excluded from screening if they were cognitively or physically incapable or terminally ill, discharged or transferred within the first 24 hours, already recruited, employed at the conducting research institute, or if they had highly infectious diseases or insufficient language skills. Computer literacy was not required. If needed, participants received a quick introduction about handling the mobile device and assessment app. Patients screening positive for at-risk alcohol use (ie, women or men with ≥4 or ≥5 points on the Alcohol Use Disorders Identification Test [AUDIT]-Consumption) [32,33] and negative for more severe alcohol problems (ie, persons with <20 on the AUDIT) [34,35] were eligible for the PECO trial.

As described in more detail elsewhere [31], enrolment was done by research assistants. Patients who provided informed written consent to participate in the trial were asked to respond to more questions on alcohol use and motivation using the app for self-assessment and were allocated to computer-based BAI (CO), in-person BAI (PE), or assessment only (AO). A sample size of 975 participants with an allocation ratio of 2:2:1 was calculated to be sufficient to detect small intervention effects concerning reduced gram of pure alcohol use, the primary outcome of the RCT [31]. Allocation was computerized and depended on the week and ward to avoid the exchange of information between study groups. Recruitment was stopped after the intended sample size was reached within the planned recruitment time of 18 months.

### Interventions

As described in more detail elsewhere, CO and PE were designed to be comparable in terms of intervention dose and content and primarily differed in method of delivery [16,31,36].

The CO group received individually tailored feedback letters at baseline, 1, and 3 months. Based on electronic and standardized data assessment, 3 to 4-page letters were created automatically by an expert system software. The software was programmed in MS Access and handled by the research staff. For the 1-month and 3-month interventions, participants were first phoned by research assistants and asked to respond to computer-assisted telephone interviews. Afterward, the software selected text modules and graphical visualizations based on the participant’s assessment data and predefined selection rules [37]. In accordance with the transtheoretical model of intentional behavior change, feedback depended on each participant’s current motivational stage of change [38]. Participants also received normative feedback, specifically feedback on (1) their current alcohol use in comparison to others of the same gender and (2) according to theoretical constructs such as processes of change, decisional balance, and self-efficacy [39] in comparison to others in the same motivational stage. At baseline, individually tailored text modules were selected from a pool of 120 text modules. At months 1 and 3, the pool was comprised of about 270 text modules as the participants also received ipsative feedback (ie, feedback on how the participant’s current data on drinking and motivation compared to the participant’s previous data). Information on the limits of low-risk drinking was provided at all time points [40]. The letters were then handed or sent out by research assistants along with a stage-matched self-help manual. Of the CO participants, 89% (345/387) received at least two feedback letters, and 72% (280/387) received all three feedback letters [16].

The PE group received in-person counseling at baseline (face-to-face on the ward) and 1 and 3 months later (via telephone). Counseling was delivered by research staff trained in motivational interviewing [41] techniques and supervised on a regular basis. Like CO, PE was stage-matched and included normative and ipsative feedback on alcohol use and theoretical constructs and information on the limits of low-risk drinking. Counselors received a one-page manual, including the same computer-generated feedback information as the letters used in CO, to ensure comparability. Over 3 months, PE participants received a total of 35 minutes (median) of counseling, with 83% (292/354) of them being counseled over at least two consultations and 54% (191/354) over three consultations. PE was delivered with acceptable adherence to motivational interviewing [16,31].

Participants in the AO group received minimal assessment at baseline (including sociodemographics, alcohol use, and motivational stage) and were not contacted at months 1 and 3.

### Measures

The outcome in this study was grams of pure alcohol consumed per day. At baseline and at all follow-ups, grams per day were assessed by 2 questions concerning the previous month. The frequency question (“In [month], how often did you have an alcoholic drink?”) included 5 response categories: never (0), once (1), 2 to 4 times (3), 2 to 3 times per week (10), and 4 times or more per week (22). The quantity question (“In [month], how many drinks did you typically have on a drinking day?”) separately asked for the numbers of drinks containing beer (0.25 L), wine or sparkling wine (0.125 L), and spirits (0.04 L). The numbers of drinks were multiplied with their associated amount of pure alcohol (9.5 g/10.9 g/10.5 g) and summed up. A quantity-frequency product was determined, divided by 30.5, and rounded.

Moderators were assessed at baseline. Education was categorized as low, middle, and high levels. Categorization was derived from the assessment of different types of school education in Germany. Participants with 9 or fewer years of schooling were allocated to low education, participants with 10 to 11 years to middle education, and those with 12 or more years to high education. Six participants, reporting to be still in school, were allocated to high education. Employment status was differentiated between employed and unemployed participants. Categorization was derived from the assessment of 2 questions: (1)“Are you currently employed?” with two response options (yes/ no) and (2) among participants who responded “no” were asked which of 6 response options applied (unemployed, pupil, college student, retired, housewife or house-husband, or other). The category “employed” included participants responding “yes” in the first question and participants providing any response other than “unemployed” in the second question to investigate the effect of actual unemployment.

Covariates included gender, age, medical department, self-rated health assessed by the single-item (ie, “Would you say your health in general is: excellent, very good, good, fair, or poor?” [42]). Mental health was assessed by the 5-item Mental Health Inventory [43,44], specifically having a partner (yes, including being married, or no), the number of cigarettes per day, alcohol problem severity assessed by the AUDIT [35], and motivational stage of change measured by a 4-item staging algorithm [16].

### Follow-Ups

Follow-ups were conducted between August 2011 and November 2014. All trial participants were followed-up 6, 12, 18, and 24 months after baseline, primarily via computer-assisted telephone interviews. Interviewers were blinded to group allocations; some of them were involved in sample recruitment 12 to 24 months earlier. Incentives were paid before (month 12: self-selected 5€ voucher) or after participation (months 6, 18, and 24: 10, 15, and 20€ voucher, respectively). An average currency exchange rate of €1 = US $1.34 was applicable during this time.

### Statistical Analysis

Data were analyzed using Mplus version 7.31(Muthén and Muthén) [45]. Two latent growth models were used to test differential BAI effects on alcohol use per day. Latent growth models afford to reflect nonlinearity and heterogeneity in the outcome growth trajectory and to handle incomplete data properly [46]. In this study, a maximum likelihood estimator with robust standard errors using numerical integration was chosen. Thus, both models were estimated under a missing at random [47] assumption using all available data and including all participants regardless of attrition. Repeated measures of alcohol per day were treated as indicators of latent growth factors that represented the alcohol growth trajectory over 24 months. As data were characterized by a large proportion of zeros with the remaining values being highly positively skewed, alcohol use per day was regressed on the growth factors using a negative binomial model. To handle nonlinearity, the model included 3 growth factors (intercept, linear, and quadratic growth factor). The variance of the quadratic growth factor was fixed to zero.

Interaction terms between the study groups and the two moderator variables (school education and employment status) were included as predictors of the growth factors to test differences in the efficacy of CO and PE. If rescaled likelihood ratio tests indicated significantly improved model fit due to the inclusion of the interaction terms, moderator level-specific net changes in alcohol use were calculated. Net changes were given in incidence rate ratios (IRRs), indicating study group differences in the percentage change in alcohol use per day between baseline and follow-up at 6, 12, 18, and 24 months, respectively. The 24-month follow-up was considered the primary time-point of interest. *P* values below .05 were considered statistically significant. Both analyses were adjusted for all baseline covariates reported above and for the remaining moderator variable.

The adjustment for the medical department also took into account potential clustering effects. Different from common cluster-randomized trials, no severe loss of power was expected: (1) all wards provided participants for each study group and (2) with the large number of 140 clusters and the small average number of 7 participants per cluster, only a small design effect (if at all) was expected [48].

## Results

### Study Sample at Baseline

**Figure 1:**
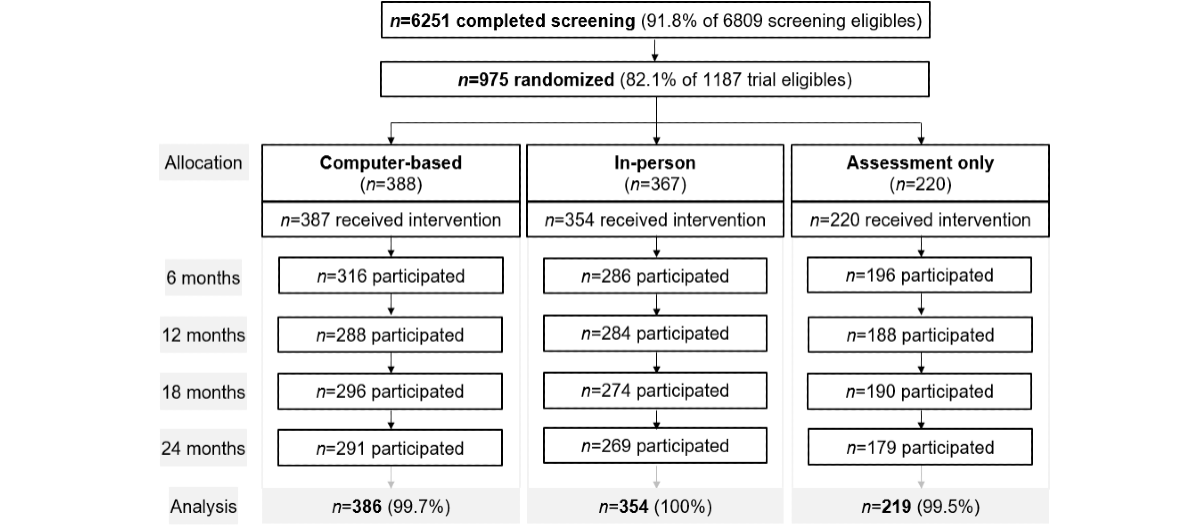
Participant flow by study group.

Of the 6809 patients eligible for screening, 6251 (92%) completed screening (Figure 1). Of the 1187 patients who screened positive for at-risk alcohol use but negative for more severe alcohol problems, 975 (82%) participated in the trial, and 961 (81%) received their allocated intervention. Follow-up participation rates were 83% (798/961) at month 6, 79% (760/961) at month 12, 79% (760/961) at month 18, and 77% (739/961) at month 24. For a detailed CONSORT flow chart, please see elsewhere [16,31]. Two participants (0.2%), 1 with missing baseline covariate data and 1 with unreasonably high alcohol data, were excluded from the analysis.

As described in more detail elsewhere [16,31], the final sample (N=959) comprised of 719 (75%) men and 240 (25%) women, with a mean age of 40.9 years (SD 14.1). Among the participants, 190 (20%), 532 (55%), and 237 (25%) had low, middle, and high levels of education, respectively. Participants consumed on average 15.2 g of pure alcohol per day (SD 19.8) at baseline. As depicted in Table 1, a total of 136 (14%) participants were unemployed, and 823 (86%) were employed, also including 96 (12%) retired persons, 61 (7%) college students or pupils, and 41 (41%) others (eg, housewives or house-husbands). Nonparticipants were older and had lower levels of education but did not differ significantly concerning any of the other characteristics [16].

**Table 1:** Moderator characteristics at baseline stratified by study group (N=959).

Moderators		Computer-based intervention (n=386)	In-person intervention (n=354)	Assessment only (n=219)
**Level of education, n (%)**				
	Low	84 (21.7)	60 (16.9)	46 (21.0)
	Middle	211 (54.7)	207 (58.5)	114 (52.1)
	High	91 (23.6)	87 (24.6)	59 (26.9)
**Employment status, n (%)**				
	Unemployed	65 (16.8)	37 (10.5)	34 (15.5)
	Employed	321 (83.2)	317 (89.5)	185 (84.5)

### Moderation Analyses

Rescaled likelihood ratio tests indicated that model fit was not significantly improved by the inclusion of interaction terms between the study group and level of education (*P*=.98). Model fit was significantly improved by including study group x employment status interactions (*P*=.04). These findings are described in more detail.

**Figure 2:**
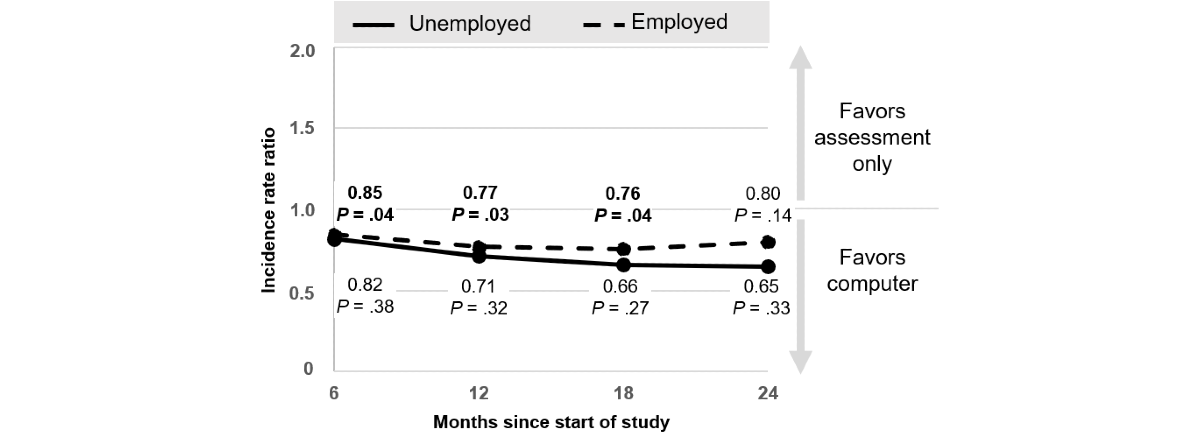
Effects of the computer-based intervention versus assessment only by employment status.

The effect of CO versus AO by employment status is depicted in Figure 2. Among employed participants, those who received CO reported significantly greater drinking reductions up to month 18 than those who received AO (IRR 0.76, 95% CI 0.58-0.99; *P*=.04). Among unemployed participants, IRRs were comparable but not statistically significant (*P*s≥.27). The efficacy of CO did not differ significantly between employed and unemployed participants (*P*s≥.66; Table 2).

**Table 2:** Net changes in alcohol use in employed versus unemployed patients (n=959).^a^

	CO^b^ versus AO^c^			PE^d^ versus AO				PE versus CO		
	IRR^e^	95% CI	*P*	IRR	95% CI	*P*	IRR		95% CI	*P*
Month 0 to 6	0.97	0.60-1.56	.90	0.58	0.35-0.95	.03	0.60		0.38-0.96	.03
Month 0 to 12	0.92	0.45-1.89	.83	0.44	0.21-0.94	.03	0.48		0.24-0.96	.04
Month 0 to 18	0.87	0.39-1.93	.73	0.44	0.18-1.06	.07	0.50		0.23-1.10	.09
Month 0 to 24	0.81	0.32-2.04	.66	0.57	0.19-1.69	.31	0.70		0.27-1.81	.46

^a^Adjusted for gender, age, having a partner, school education, medical department, self-rated health, smoking, alcohol use problem severity, and motivational stage of change.

^b^CO: computer-based intervention.

^c^AO: assessment only.

^d^PE: in-person intervention.

^e^IRR: incidence rate ratio.

**Figure 3:**
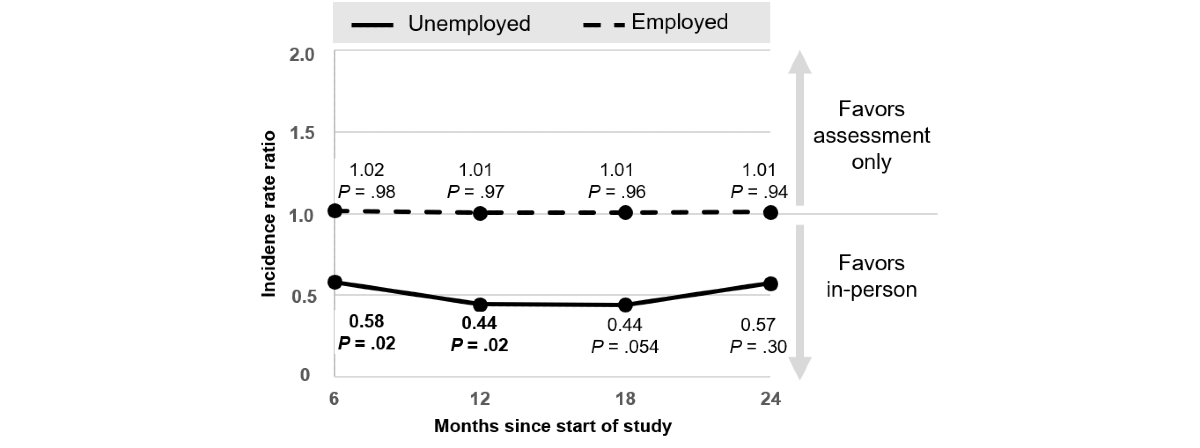
Effects of the in-person intervention versus assessment only by employment status.

The effect of PE versus AO by employment status is depicted in Figure 3. Among unemployed participants, those who received PE reported significantly greater drinking reductions up to month 12 than those who received AO (IRR=0.44, 95% CI 0.22-0.90; *P*=.02). The difference was marginally significant at month 18 (IRR=0.44, 95% CI 0.19-1.02; *P*=.054) and nonsignificant at month 24 (*P*=.30). Among employed participants, no statistically significant differences were found (*P*s≥.94). As depicted in Table 2, unemployed participants reported significantly greater drinking reductions following PE versus AO than employed participants up to month 12 (IRR 0.44, 95% CI 0.21-0.94; *P*=.03). This difference was marginally significant after 18 months (IRR 0.44, 95% CI 0.18-1.06; *P*=.07) and nonsignificant after 24 months (IRR 0.57, 95% CI 0.19-1.69; *P*=.31).

**Figure 4:**
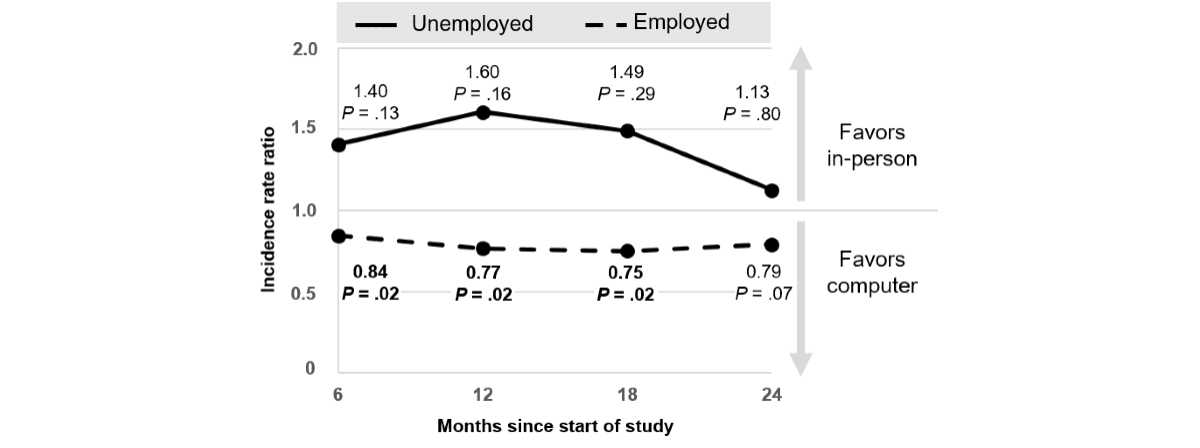
Comparative effect of computer-based versus in-person intervention by employment status.

The effect of PE versus CO by employment status is depicted in Figure 4. Among employed participants, those who received CO reported significantly greater drinking reductions up to month 18 than those who received PE (IRR 0.75, 95% CI 0.59-0.95; *P*=.02). The difference was marginally significant at month 24 (IRR 0.79, 95% CI 0.61-1.02; *P*=.07). Among unemployed participants, differences between PE and CO were not statistically significant (*P*s≥.13). As depicted in Table 2, up to month 12, unemployed participants reported significantly greater drinking reductions following PE versus CO than employed participants, while the latter rather benefitted from CO than from PE (IRR 0.48, 95% CI 0.24-0.96; *P*=.04). This difference was marginally significant after 18 months (IRR 0.50, 95% CI 0.23-1.10; *P*=.09) and not significant after 24 months (IRR 0.70, 95% CI 0.27-1.81; *P*=.46).

## Discussion

### Overview

This was the first study on the moderating effects of education and employment status on the efficacy and on the comparative efficacy of in-person versus computer-based delivered BAI. It revealed three encouraging findings. Firstly, the efficacy of computer-based BAI was neither moderated by the patients’ level of education nor by their employment status. Secondly, in-person BAI had a greater impact on reduced drinking up to month 12 in unemployed versus employed patients. Thirdly, the short-term superiority of in-person BAI over computer-based BAI in unemployed patients and of computer-based BAI over in-person BAI in employed patients was no longer significant after 2 years.

### Principal Results and Comparison With Prior Work

The finding that BAI efficacy was not moderated by the level of education is in line with previous reviews showing that once the participants had been recruited, there is no difference in effect [17,21]. While previous studies have often been limited to follow-ups of 12 months or less, our findings demonstrate that comparable efficacy was also observed in the long term. Our findings also add that, although the level of education may not make a difference, other indicators of SES may.

Although after 2 years, we found no differences in efficacy for unemployed versus employed patients, in the first year, the benefits from CO and PE were significantly reversed, indicating that unemployed patients may benefit sooner (ie, within the first year) from in-person delivered BAI, while employed patients may benefit sooner from computer-based feedback. Although these differences attenuate over time, an earlier onset of behavior change may also have other positive consequences for patients, such as earlier reduction of adverse consequences from drinking and earlier improvement of quality of life. Until now, employment status has only rarely been investigated as a moderator of behavior change interventions in general [18].

We may only speculate on why a moderation effect was found for employment status but not for school education. It is possible people with current or particularly heavy strain (as unemployment is likely to be) especially appreciate in-person conversations characterized by compassion, acceptance, partnership, and evocation as transported by motivational interviewing [49], or unemployed people especially appreciate in-time conversations also when they are more time-consuming as they provide the opportunity for answering questions. In contrast, employed people may especially appreciate the independence from the time that may be involved with computer-based feedback. However, in line with other findings on moderating effects as found in this same RCT [50,51], these findings suggest that in-person interventions may not be completely replaceable, particularly for persons with a greater strain who may require in-person rather than computer-based BAI to achieve BAI benefit as soon as possible.

Concerning the question of whether alcohol screening and BAI has at least a neutral social equity impact, the reach of the intervention investigated must also be considered. Although our approach resulted in a significantly lower reach of patients with low levels of education [16], overall reach was satisfying: 81% (961/1187) of the total target population and 79% (723/907) of those with low levels of education had been reached with our recruitment strategy. Lower-effort recruitment results in much larger selection and discrepancies. For example, a large-scale population-based intervention study in Denmark reached 53% of the total target population and 43% of those with low education [52]. With proactive recruitment, as used in our study, the extent of selectivity and discrepancy can be diminished to a great extent but may not be excluded completely. Any self-selection may result in the participation of the “(rather) healthy well-educated,” and nonsystematic selection may be driven by socially-unfavorable selection mechanisms, such as stigma. For example, although a population survey in England revealed that general practitioners approached low SES patients twice as likely as high SES patients for BAI, the selection mechanism was highly selective as less than 1 in 10 participants who would have met the eligibility criteria were approached to begin with [53].

In light of all findings on reach and on the moderators of efficacy from this RCT, we may conclude that proactive selection (ie, systematic alcohol screening) and BAI has the potential to have at least a neutral social equity impact. Equity impact may be optimized by providing computer-based BAI to the vast majority of patients with lower strain (eg, to employed patients) and by providing in-person BAI to the minority of patients with heavier strain (eg, to unemployed patients). To improve the reach of low SES people and to improve the cost-efficiency of BAI, the implementation of screening and BAI in social settings such as job agencies has been found to be promising [54].

### Strengths and Limitations

The study provides several strengths. First, the findings are based on a sample of general hospital patients representing 81% (959/1187) of the eligible patients with at-risk alcohol use. Second, the investigation of 2 indicators of SES, including employment status, which has rarely been investigated as a moderator of intervention efficacy [18], provided the opportunity to obtain a more detailed picture of the role different indicators may play in BAI efficacy. Third, the BAIs tested were theory-based, adequately delivered, and intervention retention was high [16]. The finding that intervention retention was particularly high in those receiving computer-based feedback is encouraging and is discussed in more detail elsewhere [16]. Fourth, the 4 follow-ups from 6 to 24 months provided the opportunity to investigate not only short-term changes as usual but also long-term changes by SES groups. Monetary incentives were used to reduce selection bias at follow-ups, resulting in satisfactory follow-up participation of 77%-83%. It appears unlikely that incentives have distorted study results as they were provided to participants at follow-ups only, independent of the study group, individual intervention retention, and behavior change. And fifth, latent growth modeling allowed the capture of individual differences in change over 5 time points to depict nonlinear trajectories of change and include all baseline participants in the analysis, regardless of their adherence to intervention or follow-up.

Several limitations are to be noted. First, it must be acknowledged that the RCT was powered to detect treatment effects in the total sample rather than differential treatment effects between subgroups. Therefore, potential effects did not reach statistical significance. This was particularly obvious concerning the small group of 136 unemployed participants. Second, as applies to most eHealth and BAI trials, findings are based on self-report and may be biased in terms of recall and social desirability. We cannot rule out that, as a result of receiving more attention, intervention participants responded in a more socially desirable way than assessment-only participants [55]. However, alcohol self-reports offer a minorly invasive and low-cost way of obtaining alcohol use data with acceptable validity [56], particularly among persons without severe alcohol problems, as targeted in our study [57]. Third, as also applies to most eHealth trials, participants were not blinded. Fourth, findings may be limited to those patients who agree to participate in an intervention study. Although overall reach was high, including among patients with low levels of education, nonparticipants had lower education levels and were older compared to participants [16]. The analyses were adjusted for education levels and age to account for the potential effects of these characteristics. Fifth, the generalizability of our findings may be limited to proactively recruited populations and may not apply to convenience samples given different initial characteristics in terms of problem severity and motivation to change [58].

### Conclusions

To advance the development of behavior change interventions with public health and equity impact, we, as intervention researchers, are asked to put social equity impact [5] into focus in addition to the impact of interventions on the behavioral level. To identify whether certain vulnerable members of the population benefit more or less from one or the other way of delivery, we critically investigated computer-based and in-person delivered BAIs that showed not only positive effects on reduced alcohol use but also long-term effects on health in the total sample over 2 years. The findings are encouraging with respect to reach and efficacy independent of education levels. But the study also identified that the small subgroup of unemployed patients might benefit sooner from BAI when delivered in person. These findings also highlight that, in the future, differences in intervention reach (and retention, if applicable) and efficacy or effectiveness by indicators of SES should not only be reported as descriptive measures (although it would be a good starting point) but should rather be treated as core outcome measures of behavior change interventions.

## Appendix

Multimedia Appendix 1 CONSORT eHEALTH Checklist (V 1.6.1).

## Abbreviations

AO: assessment only
AUDIT: Alcohol Use Disorder Identification Test
BAI: brief alcohol intervention
CO: computer-based intervention
IRR: incidence rate ratio
PE: in-person intervention
RCT: randomized controlled trial
SES: socioeconomic status
